# Physical activity, sedentary behavior, and metabolic-reproductive health in women with PMOS (PCOS): a narrative review of mechanisms, clinical evidence, and individualized intervention strategies

**DOI:** 10.3389/fendo.2026.1797826

**Published:** 2026-08-10

**Authors:** Xinjiang Huang, Zhi Chen, Liyi Zhang, Jie Li

**Affiliations:** 1 Department of Public Health, Dazhou Central Hospital, Dazhou, Sichuan, China; 2 Department of Gynecology, Chongqing Tradition Chinese Medicine Hospital, Chongqing, China; 3 Department of Gynecology, Dazhou Central Hospital, Dazhou, Sichuan, China; 4 Department of Clinical Research Center, Dazhou Central Hospital, Dazhou, Sichuan, China

**Keywords:** individualized intervention, metabolic complications, physical activity, polyendocrine metabolic ovarian syndrome (PMOS), sedentary behavior

## Abstract

Polyendocrine Metabolic Ovarian Syndrome (PMOS) is a common endocrine-metabolic disorder in reproductive-aged women, characterized by clinical heterogeneity and an increased susceptibility to insulin resistance, type 2 diabetes mellitus, dyslipidemia, non-alcoholic fatty liver disease, and cardiometabolic risk. Although PMOS is often recognized through reproductive manifestations such as menstrual irregularity, ovulatory dysfunction, hyperandrogenism, and infertility, metabolic dysfunction is closely linked to reproductive outcomes through effects on androgen excess, ovulatory function, oocyte competence, and the intrauterine metabolic environment. Physical activity, structured exercise, and sedentary behavior are modifiable lifestyle factors that may influence both metabolic and reproductive health in PMOS. This narrative review synthesizes current evidence on the associations among physical activity, sedentary behavior, and metabolic complications in women with PMOS. We summarize interconnected mechanisms involving insulin signaling, adipose tissue dysfunction, chronic low-grade inflammation, oxidative stress, mitochondrial function, and gut microbiota in women with PMOS, while distinguishing evidence from observational studies, clinical intervention trials, and mechanistic or preclinical research. We also review clinical evidence on exercise modalities, including aerobic exercise, resistance training, combined training, and high-intensity interval training, as well as emerging strategies to reduce or interrupt prolonged sedentary time. Available evidence supports physical activity and structured exercise as important components of PMOS management, particularly for improving insulin sensitivity, body composition, cardiometabolic risk markers, and, in some studies, menstrual cyclicity and ovulatory function. However, findings vary according to baseline BMI, hyperandrogenic phenotype, intervention type, intensity, duration, adherence, and co-interventions such as diet or pharmacotherapy. Evidence regarding sedentary behavior remains more limited and is largely observational. Therefore, causal interpretations should be made cautiously. Finally, this review discusses individualized, patient-centered intervention strategies and identifies research gaps, including long-term randomized controlled trials, reproductive and cardiometabolic hard outcomes, combined lifestyle-pharmacological approaches, and precision medicine frameworks for women with PMOS.

## 1 Introduction

Polyendocrine Metabolic Ovarian Syndrome (PMOS), formerly known as polycystic ovary syndrome (PCOS), affects 10-13% of reproductive-aged women worldwide and is one of the most prevalent endocrine-metabolic disorders in this population (1, 2). PMOS is characterized by substantial clinical heterogeneity, including menstrual irregularities, ovulatory dysfunction, clinical or biochemical hyperandrogenism, polycystic ovarian morphology, and infertility (3, 4). The diagnostic criteria for PMOS have evolved from the 1990 NIH criteria to the 2003 Rotterdam criteria, which remain widely used internationally. According to the Rotterdam criteria, PMOS is diagnosed when at least two of the following three features are present, after exclusion of related disorders: clinical or biochemical hyperandrogenism, oligo-ovulation or anovulation, and polycystic ovarian morphology on ultrasound (5). Based on these criteria, PMOS can be classified into four phenotypes: Phenotype A (concurrent hyperandrogenism, ovulatory dysfunction and polycystic ovarian morphology), Phenotype B (hyperandrogenism plus ovulatory dysfunction without polycystic ovarian morphology), Phenotype C (hyperandrogenism plus polycystic ovarian morphology without ovulatory dysfunction), and Phenotype D (ovulatory dysfunction combined with polycystic ovarian morphology in the absence of hyperandrogenism) (5, 6). Phenotypes A and B are categorized as “classic PCOS” with the highest metabolic and reproductive risks, whereas the metabolic risk profile of Phenotypes C and D may be lower but remains heterogeneous across populations (6–8). PMOS is not merely a reproductive disorder but is increasingly recognized as a complex metabolic disease (4, 9). A substantial proportion of women with PMOS exhibit insulin resistance, which exacerbates hyperandrogenism and metabolic abnormalities across the body mass index (BMI) spectrum (4, 10). Hyperinsulinemia can amplify ovarian androgen production and reduce sex hormone-binding globulin (SHBG) levels. These mechanisms collectively link metabolic dysfunction with hyperandrogenism, ovulatory disturbance, and reproductive impairment (10–12). Women with PMOS also have increased risk of type 2 diabetes mellitus (T2DM), metabolic syndrome, non-alcoholic fatty liver disease (NAFLD), and cardiovascular disease (CVD) (13–16).

Physical activity, structured exercise, and sedentary behavior are modifiable lifestyle factors that are closely related to metabolic health. Epidemiological evidence suggest that women with PMOS report insufficient physical activity, prolonged sedentary time, and suboptimal dietary patterns, although findings vary across population and measurement methods (17, 18). These behaviors are associated with weight gain, insulin resistance, dyslipidemia, and other metabolic abnormalities (13, 17–23), but observational data cannot fully exclude residual confounding. International guidelines recommend lifestyle management, including physical activity and exercise, as a first-line component of PMOS care (13). Clinical studies indicate that aerobic exercise, resistance training, combined lifestyle interventions, and high-intensity interval training may improve body weight, insulin sensitivity, lipid profiles, cardiorespiratory fitness, menstrual cyclicity, ovulatory function, and psychological well-being in some women with PMOS (13, 24–27). However, the magnitude and durability of benefit differ across studies, partly because of variation in intervention type, intensity, frequency, duration, supervision, adherence, baseline BMI, and concurrent dietary or pharmacological interventions. The relevance of physical activity and sedentary behavior to PMOS therefore extends beyond metabolic complications alone. Metabolic dysfunction, chronic low-grade inflammation, adipose tissue dysfunction, and androgen excess may interact with reproductive processes, including follicular development, ovulatory function, endometrial receptivity, and fertility-related outcomes (28–30). Conversely, improvement in metabolic health through lifestyle intervention may support reproductive management, although evidence for hard reproductive endpoints such as live birth remains less consistent than evidence for intermediate metabolic outcomes (13, 27). This metabolic-reproductive interface is particularly relevant for interpreting lifestyle intervention studies in PMOS and for designing future trials that include both metabolic and reproductive endpoints.

Interpretation remains challenging because existing studies vary in PMOS definitions and phenotypes, participant metabolic profiles, exposure assessment, intervention design, and outcome measures. Mechanistic explanations require caution because evidence comes from mixed sources, including preclinical, observational, and clinical studies. Clinical translation is further constrained by limited individualized guidance, psychological distress, weight stigma, restricted access to supervised programs, and variable long-term adherence (27, 31). Accordingly, this narrative review aims to synthesize current evidence on the relationships among physical activity, sedentary behavior, and metabolic-reproductive health in women with PMOS. We first outline the search strategy and definitions used in this review, then discuss interconnected pathophysiological mechanisms, summarize observational and interventional evidence, compare exercise modalities and sedentary behavior reduction strategies, and consider individualized intervention approaches based on phenotype, metabolic profile, patient preferences, and practical barriers. By distinguishing associations from causal evidence and highlighting areas of uncertainty, this review seeks to provide a clinically relevant framework for lifestyle management and future research in women with PMOS.

## 2 Search strategy, methodology, and definitions

### 2.1 Search strategy and information sources

This manuscript is structured as a narrative review aiming to synthesize current evidence on the interplay between physical activity, sedentary behavior, and metabolic health in women with PMOS. To ensure a comprehensive and transparent literature base, we conducted a focused search of PubMed, Web of Science, and Scopus for articles published from January 1980 to June 2026. The search strategy combined terms related to exposures (“physical activity,” “exercise,” “sedentary behavior,” “sedentary time”), outcomes (“metabolism,” “insulin resistance,” “glucose metabolism,” “lipid metabolism,” “obesity,” “cardiovascular,” “non-alcoholic fatty liver disease,” “reproductive outcomes,” “ovulation,” “fertility”), and context (“polycystic ovary syndrome,” “PCOS,” “women’s health,” “public health,” “guidelines”). The initial screening was based on titles and abstracts to identify relevant original research articles, systematic reviews, and meta-analyses. The final selection was made based on full-text review, prioritizing high-impact clinical trials and mechanistic studies. Given the heterogeneity of study designs and outcomes in this field, this review adopts a narrative synthesis approach rather than a quantitative meta-analysis to provide a broad and clinically relevant perspective.

### 2.2 Definitions of physical activity, exercise, and sedentary behavior

According to the World Health Organization (WHO) (32), sedentary behavior is defined as any waking activity characterized by an energy expenditure of ≤1.5 metabolic equivalents while in a sitting, reclining, or lying posture. This includes common activities such as office work, driving, and television viewing, and also applies to individuals who are unable to stand, including wheelchair users. Operationally, this behavior is assessed through self-reported sitting time (across leisure, occupational, and total contexts), screen time, or objective measures of low movement via devices. In contrast, physical activity is broadly defined as any bodily movement generated by the contraction of skeletal muscles those results in a substantial increase over resting energy expenditure. Within this broader category, exercise is distinguished as a subcategory that is planned, structured, repetitive, and performed deliberately with the specific objective of improving or maintaining one or more components of physical fitness. In practice, the terms “exercise” and “exercise training” are often used interchangeably, typically referring to leisure-time physical activity undertaken primarily for health, performance, or fitness enhancement.

## 3 Mechanistic links physical activity, sedentary behavior, and metabolic-reproductive dysfunction in PMOS

### 3.1 Metabolic-reproductive dysfunction in PMOS: an integrated pathophysiological framework

PMOS-related metabolic dysfunction arises from an interconnected network of endocrine, metabolic, inflammatory, neuroendocrine, and genetic factors rather than from isolated pathways Hyperandrogenism and insulin resistance are central and mutually reinforcing features of this network (29, 33). Androgen excess may affect peripheral metabolic tissues, including adipose tissue, liver, pancreas, and skeletal muscle, and has been linked to impaired insulin signaling, altered lipid metabolism and visceral adiposity. Conversely, insulin resistance and compensatory hyperinsulinemia can stimulate ovarian androgen production and reduce sex hormone-binding globulin (SHBG), thereby amplifying hyperandrogenism and contributing to ovulatory dysfunction (10, 29, 34). Importantly, in women with PMOS, insulin resistance may be present in both lean individuals and those with overweight or obesity, although excess adiposity can further intensity metabolic abnormalities (10, 35, 36).

Adipose tissue dysfunction is another key component linking PMOS to metabolic and reproductive abnormalities. Reported alterations include impaired adipogenesis, dysregulated lipolysis, altered adipokine and cytokine secretion, mitochondrial dysfunction, oxidative stress, endoplasmic reticulum stress, and chronic low-grade inflammation (30, 37, 38). Reduced insulin-mediated glucose uptake, partly related to altered glucose transporter-4 (GLUT-4) expression and insulin signaling, may contribute to systemic insulin resistance (39). Adipose tissue inflammation may also interact with androgen excess and metabolic dysfunction, indicating that inflammation adiposity, and insulin resistance should be interpreted as overlapping processes rather than independent mechanisms (30, 38, 40).

Mitochondrial dysfunction and oxidative stress have also been implicated in PMOS pathophysiology. Evidence from studies assessing leukocyte mitochondrial function and circulating oxidative stress markers suggests impaired oxidative metabolism, increased ROS production, altered antioxidant capacity, and associations with insulin resistance in women with PMOS (41–43). However, the strength of evidence varies across tissues and study designs, and some mechanistic findings are derived from preclinical or cellular models. Therefore, these abnormalities should be interpreted as plausible biological pathways that may contribute to PMOS pathophysiology rather than as definitive causal mechanisms applicable to all women with PMOS.Neuroendocrine dysregulation further connects metabolic and reproductive features of PMOS. Altered hypothalamic-pituitary-ovarian (HPO) axis activity, including changes in gonadotropin-releasing hormone pulsatility and luteinizing hormone secretion, can disrupt ovarian steroidogenesis and ovulatory function (44–46). Central metabolic circuits, sympathetic nervous system activity, gut hormones, and the gut microbiota may additionally influence appetite regulation, energy balance, inflammation, insulin sensitivity, and androgen production (45–48). Emerging evidence also suggests that genetic susceptibility, epigenetic regulation, and non-coding RNAs may contribute to the heterogeneity of PMOS phenotypes and metabolic risk (49, 50).

**Figure 1:**
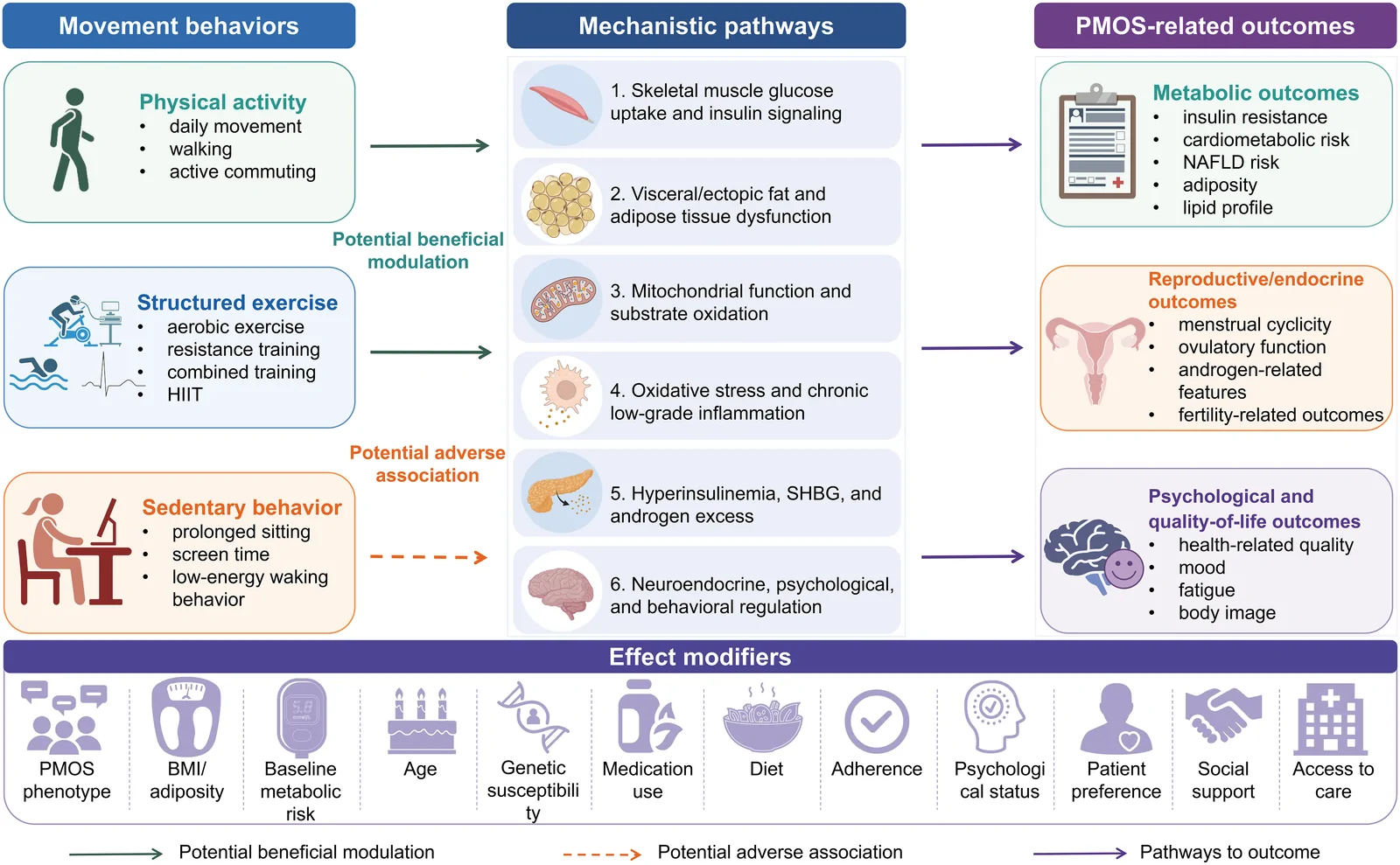
Movement behaviors, mechanisms, and outcomes in polyendocrine metabolic ovarian syndrome (PMOS). Physical activity, structured exercise, and sedentary behavior may influence PMOS-related metabolic, reproductive/endocrine, and psychological outcomes through insulin-related, adipose, mitochondrial, inflammatory, hormonal, and neuroendocrine-behavioral pathways. These associations may be modified by PMOS phenotype, body mass index (BMI)/adiposity, baseline metabolic risk, age, genetics, medication use, diet, adherence, psychological status, patient preference, social support, and access to care. SHBG, sex hormone-binding globulin; NAFLD, non-alcoholic fatty liver disease. Partial elements were created with BioRender.com (Created in BioRender. Jie, L. (2026) https://BioRender.com/iwkohh9). Additional graphical elements were constructed using Microsoft PowerPoint.

In summary, PMOS-related metabolic-reproductive dysfunction is best understood as a dynamic network involving hyperandrogenism, insulin resistance, adipose tissue dysfunction, mitochondrial dysfunction, oxidative stress, chronic low-grade inflammation, neuroendocrine dysregulation, and genetic or epigenetic susceptibility. These pathways interact bidirectionally and may help explain why metabolic abnormalities in PMOS are closely linked to menstrual irregularity, ovulatory dysfunction, fertility-related outcomes, and long-term cardiometabolic risk (**Figure 1**). This integrated framework also provides a rationale for examining how physical activity, exercise, and sedentary behavior may influence PMOS-related metabolic and reproductive outcomes through multiple interacting pathways.

### 3.2 Mechanisms through physical activity and exercise improves metabolic-reproductive health in PMOS

Physical activity and structured exercise are recommended as core components of lifestyle management in PMOS (13). Their benefits are likely mediated through multiple interconnected pathways, including skeletal muscle glucose uptake, insulin signaling, mitochondrial adaptation, adipose tissue remodeling, inflammatory regulation, body composition, and neuroendocrine function (25, 26, 51–53). The strength of evidence is greatest for improvements in intermediate metabolic outcomes, whereas evidence for long-term reproductive and hard cardiometabolic outcomes remains more limited (25, 26, 54).

Skeletal muscle is a major site of insulin-stimulated glucose disposal. Exercise training can improve insulin sensitivity by increasing GLUT-4 translocation and expression, enhancing muscle glucose uptake, promoting fatty acid oxidation, and improving mitochondrial oxidative capacity (51–53). In women with PMOS, aerobic exercise, resistance training, and high-intensity interval training (HIIT) have been associated with reductions in fasting insulin and homeostatic model assessment of insulin resistance (HOMA-IR) (a measure of insulin resistance) in several intervention studies and meta-analyses, although the magnitude of benefit varies according to baseline BMI, insulin resistance status, exercise intensity, intervention duration, supervision, and adherence (25, 26, 54–58).

Exercise may also improve mitochondrial function and oxidative stress. Aerobic and resistance exercise can stimulate mitochondrial biogenesis through regulators such as peroxisome proliferator-activated receptor gamma coactivator-1α, increase antioxidant capacity, and reduce markers of oxidative damage such as malondialdehyde in some studies (51–53, 56, 59). These adaptations may contribute to improved cellular energy metabolism and insulin signaling. However, not all mechanistic evidence is PMOS-specific, and tissue-level findings from skeletal muscle, adipose tissue, or ovarian cells should be interpreted according to the experimental context (51–53).

Improvements in body composition and adipose tissue function represent another important pathway. Exercise interventions may reduce waist circumference, visceral adiposity, body fat percentage, and body mass index, particularly when combined with dietary intervention or delivered with sufficient intensity and adherence (25–27, 54, 55, 57). These changes can improve adipokine profiles, reduce ectopic lipid accumulation, and contribute to better lipid metabolism. Reported effects on total cholesterol, LDL cholesterol, triglycerides, and HDL cholesterol are generally favorable but heterogeneous across studies (25–27, 54, 57).

Exercise may also influence hormonal and reproductive outcomes, partly through improved insulin sensitivity and reduced hyperinsulinemia. Some studies suggest that structured exercise can increase SHBG, reduce circulating androgens, improve menstrual cyclicity, and support ovulatory function (60–62). Nevertheless, evidence for fertility endpoints, pregnancy outcomes, and live birth remains less consistent and is often confounded by co-interventions such as diet, weight loss, ovulation induction, metformin, or oral contraceptive use (27, 63).

Physical activity may further modulate chronic low-grade inflammation and epigenetic regulation. Clinical studies in women with PMOS have reported exercise-related changes in inflammatory markers and adipokines. Complementary evidence from animal models suggests that exercise may also influence adipose tissue gene expression, including pathways related to leptin signaling and energy metabolism (64–66). Preliminary evidence from human and animal studies suggests that exercise may induce epigenetic changes, including DNA methylation changes, that could contribute to metabolic adaptation (66–68). However, the available evidence remains limited, and findings from animal models require validation in larger PMOS-specific clinical cohorts.

Collectively, these findings suggest that physical activity and exercise improve metabolic health in women with PMOS through multiple interconnected pathways, including enhanced insulin sensitivity, greater skeletal muscle glucose uptake, mitochondrial adaptation, adipose tissue remodeling, reduced inflammation, and improved body composition. However, the extent to which these mechanistic benefits translate into clinical outcomes depends largely on the implementation of sustainable and individualized exercise interventions.

### 3.3 Mechanisms linking sedentary behavior to metabolic-reproductive dysfunction in PMOS

Sedentary behavior has emerged as a potentially relevant lifestyle factor in PMOS, although the evidence base remains substantially less developed than that for structured exercise. Most available studies are observational, and causal inference is therefore limited. Prolonged sedentary time may contribute to metabolic dysfunction through reduced skeletal muscle contractile activity, lower energy expenditure, impaired glucose handling, and adverse body composition (69, 70). In women with PMOS, these effects may interact with pre-existing insulin resistance, adipose tissue dysfunction, and hyperandrogenism (28, 71).

Higher sedentary time may be associated with poorer glucose homeostasis and greater insulin resistance in PMOS, but whether these associations are independent of adiposity, diet, sleep, and overall physical activity remains unclear (13, 18, 28). Sedentary behavior may also relate to low-grade inflammation and adipose tissue dysfunction, both of which are already common in PMOS and closely linked to obesity (72–74). Additional pathways involving oxidative stress, mitochondrial function, and gut microbiota are biologically plausible, but direct PMOS-specific evidence remains limited (75, 76).

Overall, sedentary behavior is likely to contribute to metabolic risk in PMOS, but current evidence is insufficient to define its independent mechanistic role with the same confidence as the benefits of structured exercise. Future studies using objective sedentary behavior measures and longitudinal or interventional designs are needed to clarify causality and identify clinically meaningful targets for behavioral intervention.

## 4 Clinical evidence on physical activity, exercise, and sedentary behavior in women with PMOS

### 4.1 Effects of exercise modalities on metabolic and reproductive-related outcomes

Clinical trials and systematic reviews generally support structured exercise as an important non-pharmacological component of PMOS management. Across available studies, the most consistent benefits are observed for cardiometabolic and fitness-related intermediate outcomes, particularly insulin resistance, body composition, waist circumference, and cardiorespiratory fitness, with some evidence for improvements in lipid profiles and inflammatory markers (25, 31, 57, 77). By contrast, effects on androgen-related, menstrual, ovulatory, and fertility outcomes are less consistent and remain limited by small sample sizes, heterogeneous exercise protocols, differences in PMOS phenotypes, and the multifactorial nature of reproductive dysfunction in PMOS (25–27, 77).

Several factors appear to influence the clinical response to exercise in women with PMOS. Exercise intensity, weekly exercise volume, intervention duration, supervision, adherence, baseline BMI, metabolic phenotype, and concurrent dietary or pharmacological interventions may all affect observed outcomes (25, 77). Systematic reviews and meta-analyses suggest that moderate-to-vigorous exercise and programs achieving sufficient weekly exercise volume are more consistently associated with improvements in HOMA-IR, BMI, waist circumference, and cardiorespiratory fitness (13, 26). However, current evidence does not establish one universally optimal exercise prescription, as studies differ substantially in intervention design, control conditions, outcome measures, and follow-up duration.

Aerobic exercise is one of the most commonly studied exercise modalities in women with PMOS. Interventions typically include brisk walking, running, cycling, or treadmill-based training, often performed at least three times per week for 30–60 minutes per session over 8–12 weeks or longer (57, 78–81). Across clinical studies, aerobic exercise has been associated with reductions in BMI, waist circumference, fasting insulin, and HOMA-IR, as well as improved cardiorespiratory fitness; favorable changes in lipid profiles have also been reported, although less consistently (57, 77–80). These benefits appear more evident when aerobic exercise is performed at moderate-to-vigorous intensity and when adherence is adequate (57, 78, 80, 82). Some studies have also reported improvements in androgen-related markers or menstrual cyclicity after aerobic exercise, although these reproductive-related effects are less consistent than metabolic improvements (57, 77, 81, 83).

Resistance training may also be beneficial for women with PMOS, particularly with respect to body composition, muscular strength, and insulin resistance (26, 31, 58, 61). However, resistance-based interventions vary considerably in exercise selection, training frequency, load progression, and supervision. Some studies have reported reductions in fasting insulin, HOMA-IR, total testosterone, and free androgen index, together with improvements in strength and lean mass-related outcomes (58, 61). Nevertheless, the limited number of trials and variability in training protocols make it difficult to define an optimal resistance training prescription for PMOS (26, 31). Combined aerobic and resistance training may be clinically useful because it integrates cardiorespiratory and strength-related adaptations within the same intervention (26, 31, 55). Some studies have reported favorable effects on fasting insulin, HOMA-IR, blood lipids, total testosterone, and physical fitness (25, 26, 31, 55, 83). However, whether combined training is superior to aerobic or resistance training alone remains uncertain, because available trials differ in total exercise dose, intervention duration, supervision, and co-interventions (25, 26, 31).

HIIT has received increasing attention as a time-efficient exercise strategy for women with PMOS. Randomized controlled trials suggest that HIIT may improve insulin-related outcomes and cardiorespiratory fitness over relatively short intervention periods, commonly around 10–16 weeks, with some studies also reporting favorable changes in body composition and endothelial function (26, 58). Its lower time requirement may be attractive for some patients, particularly those who identify lack of time as a barrier to exercise. However, HIIT should not be viewed as universally suitable. Baseline fitness, obesity severity, musculoskeletal limitations, psychological readiness, tolerability, safety, and long-term adherence should all be considered before recommending high-intensity protocols (26, 82, 84).

Beyond modality-specific effects, exercise may improve several cardiometabolic risk markers in women with PMOS. Trials and reviews have reported reductions in total cholesterol, LDL cholesterol, triglycerides, waist circumference, and some inflammatory markers, although effects on HDL cholesterol appear less consistent (25, 31). Some studies have also described favorable changes in blood pressure and inflammatory markers, including C-reactive protein, following exercise interventions in women with PMOS (31, 57, 85). These findings are clinically relevant given the adverse cardiometabolic profile frequently observed in women with PMOS. However, most exercise trials are relatively short and are not designed to evaluate hard cardiovascular outcomes, such as incident diabetes, cardiovascular events, or long-term morbidity. This broader cardiovascular perspective is particularly important because cardiovascular risk profiles and preventive strategies may differ between women and men, and sex-specific considerations are increasingly emphasized in cardiovascular medicine (86). Future studies in PMOS should therefore assess longer-term cardiovascular endpoints in addition to short-term metabolic markers, ideally within a sex-informed preventive framework.

Reproductive-related outcomes have been reported less consistently than metabolic outcomes (85). Some studies have observed improvements in menstrual cyclicity and selected reproductive hormonal markers, including SHBG, total testosterone, and free androgen index (55, 81, 83). These changes may be more likely when exercise is accompanied by improvements in insulin resistance, body composition, or weight-related outcomes. However, evidence for fertility, pregnancy, and live birth outcomes remains limited (55, 87, 88). Interpretation is also complicated by concurrent weight loss, dietary intervention, ovulation induction, metformin, oral contraceptive use, and other pharmacological treatments. Future trials should therefore include reproductive endpoints alongside metabolic outcomes to determine whether exercise-induced changes translate into clinically meaningful reproductive benefits (89).

Overall, structured exercise appears beneficial for several intermediate metabolic outcomes in women with PMOS, particularly insulin resistance, body composition, waist circumference, lipid-related markers, inflammatory markers, and cardiorespiratory fitness. Aerobic exercise, resistance training, combined training, and HIIT may all have clinical value, but current evidence does not support one universally optimal modality or prescription. Exercise recommendations should therefore be individualized according to clinical phenotype, baseline metabolic risk, reproductive goals, patient preference, safety, access to supervision, and the likelihood of long-term adherence.

### 4.2 Effects of sedentary behavior on metabolic and reproductive-related outcomes in PMOS

Compared with exercise interventions, the evidence on sedentary behavior in PMOS is less mature and is mainly derived from cross-sectional or observational studies. Available studies suggest that longer sedentary time may be associated with less favorable metabolic characteristics, particularly higher adiposity and central adiposity, in women with PMOS. Associations with insulin resistance and other cardiometabolic risk markers have also been reported, but these findings are less consistent and are difficult to separate from the influence of total physical activity, adiposity, and related lifestyle factors (90–92). In contrast, direct evidence linking sedentary behavior to reproductive-related outcomes in PMOS, such as menstrual cyclicity, ovulation, androgen excess, fertility, pregnancy, or live birth, remains very limited. Because sedentary behavior is closely correlated with diet, sleep, psychological distress, occupational factors, total physical activity, and adiposity, causal conclusions regarding its independent effects on metabolic or reproductive outcomes should be made cautiously (93, 94).

The most consistent observations relate to metabolic health in PMOS. A recent study applying a 24-hour movement behavior framework found that, in women with PMOS, modeled reallocation of time from sedentary behavior to moderate-to-vigorous physical activity was associated with favorable differences in cardiometabolic markers, including waist circumference, triglycerides, fasting insulin, and HOMA-IR (90). However, these findings are based on observational associations and theoretical time substitutions rather than intervention data, and therefore should not be interpreted as direct evidence that reducing sedentary time alone will produce comparable metabolic benefits in clinical settings.

Evidence for reproductive-related outcomes is considerably weaker. Few studies have specifically examined whether sedentary behavior is independently associated with menstrual cyclicity, ovulatory function, androgen-related biomarkers, fertility treatment response, pregnancy outcomes, or live birth in women with PMOS (89). At present, any relationship between sedentary behavior and reproductive dysfunction remains insufficiently characterized and, if present, may be mediated indirectly through adiposity, insulin resistance, and broader metabolic impairment rather than reflecting a clearly established independent effect.

Overall, sedentary behavior may represent a clinically relevant but still understudied component of the movement behavior profile in women with PMOS. Current evidence supports a cautious interpretation that prolonged sedentary time is associated with less favorable metabolic characteristics, whereas evidence for direct reproductive effects remains insufficient. Accordingly, reducing sedentary time should currently be viewed as a practical and low-risk adjunct to structured exercise and other lifestyle interventions, rather than an evidence-equivalent substitute for exercise. Future studies should incorporate objective measures of sedentary behavior, longitudinal designs, and intervention-based approaches to determine whether reducing total sedentary time or interrupting prolonged sitting improves clinically meaningful metabolic and reproductive outcomes in women with PMOS.

### 4.3 Combined lifestyle and pharmacological approaches

In clinical practice, exercise and sedentary behavior reduction are rarely implemented in isolation. Many women with PMOS also receive dietary intervention, combined oral contraceptives, metformin, anti-androgen therapy, ovulation induction agents, or weight-management pharmacotherapy depending on their symptoms, metabolic risk, reproductive goals, and contraindications (13, 95). This is important when interpreting exercise trials because concurrent interventions may contribute to heterogeneity in metabolic and reproductive outcomes (77, 85).

Metformin is commonly used in women with PMOS with impaired glucose regulation, higher metabolic risk, or contraindications to other therapies, and lifestyle intervention is generally recommended alongside pharmacological management rather than as a competing strategy (95). Exercise may complement metformin by improving cardiorespiratory fitness, body composition, and insulin sensitivity (77). However, the additional benefit of combining structured exercise with metformin remains incompletely defined because available studies vary in participant characteristics, intervention duration, and outcome measures (77, 85).

Combined oral contraceptives are frequently used for menstrual irregularity and hyperandrogenic symptoms, but their use should be individualized with consideration of cardiometabolic risk factors, potential contraindications, and patient priorities (95). For women using oral contraceptives, exercise and dietary management remain important for supporting metabolic health and improving fitness, body composition, and psychological well-being (77, 85). Future trials should report contraceptive and pharmacological use more consistently and examine whether medication status modifies the response to exercise or sedentary behavior interventions.

For women seeking pregnancy, lifestyle intervention may be combined with ovulation induction or other fertility treatments (95). Improvements in body composition and insulin sensitivity may support ovulatory function, but evidence for live birth or pregnancy outcomes attributable specifically to exercise remains limited (13, 85). Therefore, combined lifestyle-pharmacological strategies should be individualized according to metabolic risk, reproductive goals, medication indications, and patient preferences, with shared decision-making and long-term adherence as central considerations.

## 5 Individualized intervention strategies for physical activity and sedentary behavior in PMOS

### 5.1 Core principles of individualized management

As outlined above, PMOS encompasses a heterogeneous spectrum of reproductive, endocrine, metabolic, and psychological features (6, 95). Although the Rotterdam phenotypic classification is useful for describing this heterogeneity, it should not be applied as a rigid basis for lifestyle prescription (5). Instead, individualized management should be guided by structured phenotyping together with a broader assessment of adiposity, insulin resistance, cardiometabolic risk, reproductive goals, psychological burden, physical fitness, and access to lifestyle support (95).

Available evidence suggests that hyperandrogenic phenotypes, particularly phenotypes A and B, are more frequently associated with adverse metabolic features, including insulin resistance, central adiposity, dyslipidemia, and metabolic syndrome, whereas phenotype D may show a relatively milder metabolic profile in some cohorts (6, 8). However, these differences are not absolute, and metabolically relevant abnormalities may also occur in lean women and in those with non-hyperandrogenic phenotypes (6, 96). Therefore, phenotype may inform risk stratification, but current evidence is insufficient to support phenotype-specific exercise prescriptions (77). Accordingly, the stratified recommendations in **Table 1** are not intended to assign fixed exercise regimens to Rotterdam phenotypes. Rather, they synthesize evidence from international PMOS guidelines, general physical activity recommendations, and exercise intervention studies in PMOS, and apply these data according to clinically measurable risk factors such as adiposity, insulin resistance, cardiometabolic comorbidity, age, psychological barriers, and sedentary exposure. A comprehensive assessment should include anthropometric measures, glycemic and insulin-related indices, lipid profile, blood pressure, and, where clinically indicated, evaluation for hepatic steatosis. It should also address habitual physical activity, sedentary behavior, sleep, smoking, medication use, reproductive plans, and family history of diabetes or cardiovascular disease (13, 95).

**Table 1:** Risk-stratified considerations for exercise and sedentary behavior management in women with polyendocrine metabolic ovarian syndrome (PMOS).

Stratified groups	Key indicators	Suggested intervention	Main expected benefits
Higher metabolic-risk patients	Obesity/central adiposity, insulin resistance, impaired glucose regulation, dyslipidemia, hypertension, metabolic syndrome, low fitness; hyperandrogenic phenotypes may indicate higher risk but should not be used alone for prescription	Progressive aerobic plus resistance training; gradually progress toward 150–300 min/week moderate-intensity or 75–150 min/week vigorous-intensity aerobic activity, plus resistance training ≥2 days/week; use low-impact options when needed	Improved insulin sensitivity, body composition, cardiorespiratory fitness, lipid profile, and cardiometabolic risk
Lower apparent metabolic-risk or lean patients	Normal BMI, no overt dysglycemia/dyslipidemia/hypertension; metabolic screening still required	Follow general adult physical activity recommendations; prioritize regularity, preference, sustainability, and fitness improvement rather than weight loss alone	Improved fitness, insulin sensitivity, menstrual function, psychological well-being, and quality of life
Adolescents with PMOS or suspected PMOS	Pubertal stage, menstrual irregularity, hyperandrogenism, obesity risk, body image or psychological concerns	Age-appropriate physical activity; average ≥60 min/day moderate-to-vigorous physical activity, including muscle- and bone-strengthening activities ≥3 days/week; avoid weight stigma and involve family support	Healthy growth, bone development, cardiometabolic health, menstrual health, and psychological well-being
Patients with psychological distress or poor adherence	Anxiety, depression, fatigue, low self-efficacy, body image concerns, disordered eating risk, weight stigma, poor social support	Start with achievable goals; use motivational interviewing, SMART goals, self-monitoring, social support, and patient-preferred activities	Improved adherence, self-efficacy, mood, quality of life, and long-term maintenance
Patients with comorbid chronic diseases or safety concerns	Type 2 diabetes, hypertension, cardiovascular disease, severe obesity, osteoarthritis, musculoskeletal pain, or other limitations	Individualize type, intensity, and progression; consider low-impact or supervised exercise; follow disease-specific safety guidance	Safer participation, improved metabolic and functional outcomes
Sedentary or low-physical-activity patients	Prolonged sitting, low step count, low structured activity, high screen time	Interrupt sitting every 30–60 min; add standing/walking breaks, postprandial walks, step-count goals, and gradual replacement of sedentary time with light activity	Reduced sedentary exposure and potential improvement in glucose and cardiometabolic risk markers

These are risk-stratified clinical considerations rather than phenotype-specific exercise prescriptions. Current evidence does not support fixed exercise regimens based solely on Rotterdam phenotypes. Recommendations are derived from PMOS guidelines, general physical activity guidelines, PMOS exercise studies, behavioral evidence, and sedentary behavior literature.

Psychological and behavioral factors are equally important, as they influence symptom burden, engagement with care, and adherence to lifestyle intervention (97). Screening for depression, anxiety, sleep disturbance, disordered eating, and reduced quality of life may help identify barriers that require tailored support (89, 97). In this sense, individualized management should be viewed as an ongoing process that integrates phenotype, metabolic risk, psychosocial context, patient preference, and feasibility over time (13).

### 5.2 Individualized exercise and sedentary behavior management

Lifestyle management, including diet, physical activity, exercise, and behavioral support, is recommended as a first-line component of PMOS care (13). In general, women with PMOS should be encouraged to follow adult physical activity recommendations of 150–300 min/week of moderate-intensity aerobic activity or 75–150 min/week of vigorous-intensity aerobic activity, together with muscle-strengthening activities on at least 2 days/week (89, 98). When weight management is a specific therapeutic goal, higher activity volumes may be considered when feasible and safe, particularly when combined with dietary and behavioral support (99, 100). However, exercise prescription should be individualized according to baseline fitness, metabolic risk, body composition, reproductive goals, comorbidities, safety considerations, psychological barriers, and patient preference (13, 99).

Sedentary behavior reduction should be incorporated as an adjunctive target rather than a substitute for structured exercise. Practical strategies include interrupting prolonged sitting, incorporating short walking breaks, increasing light-intensity movement during daily routines, and replacing sedentary leisure with more active alternatives when feasible (26, 90, 101). Because PMOS-specific evidence on sedentary behavior interventions remains limited, these measures should be framed as pragmatic, low-risk strategies that may complement exercise and support overall metabolic health rather than as stand-alone therapies (85, 102).

For women with higher metabolic risk, including those with obesity, abdominal adiposity, marked insulin resistance, impaired glucose regulation, dyslipidemia, or hyperandrogenic phenotypes, the main intervention goals are to improve insulin sensitivity, cardiorespiratory fitness, body composition, lipid metabolism, and long-term cardiometabolic risk (26, 103). A progressive program combining aerobic and resistance exercise is generally appropriate, with gradual progression toward guideline targets (26). In women with low baseline fitness, severe obesity, musculoskeletal pain, osteoarthritis, or cardiovascular limitations, low-impact modalities such as walking, swimming, cycling, or elliptical training may improve feasibility, safety and adherence (13, 104–106).

For lean women with PMOS or those with lower apparent metabolic risk, the clinical focus should not be limited to weight loss alone. Exercise may still be prescribed to improve insulin sensitivity, cardiorespiratory fitness, menstrual regularity, psychological well-being, and quality of life (26, 27). In this context, the choice of modality should prioritize preference, sustainability, and reduction of psychological barriers (107). Home-based programs, group exercise, dance, yoga, or recreational sports may be useful when they enhance enjoyment and long-term participation, although comparative PMOS-specific evidence for superiority of any one modality remains limited (26, 108).

In adolescents with PMOS or suspected PMOS, lifestyle intervention should support healthy growth, bone development, menstrual health, cardiometabolic health, and psychological well-being (109, 110). General recommendations for children and adolescents emphasize an average of at least 60 minutes/day of moderate-to-vigorous physical activity across the week, including muscle- and bone-strengthening activities on at least 3 days/week (32, 98). Counseling should avoid weight stigma and should incorporate family support, developmentally appropriate goal-setting, and attention to body image, eating behavior, and mental health (109, 111, 112).

For women with poor adherence, fatigue, anxiety, depression, low self-efficacy, or limited social support, exercise prescriptions should prioritize achievable starting points, gradual progression, patient-preferred modalities, and barrier reduction rather than rapid dose escalation (13, 27). Emotional distress, body image concerns, disordered eating, or weight stigma should be considered when tailoring exercise goals and referral pathways (113, 114).

In women with comorbid chronic diseases, including T2DM, hypertension, cardiovascular disease, osteoarthritis, or severe obesity, exercise type, intensity, and progression should be adapted according to disease-specific guidelines and safety considerations (115, 116). Medical evaluation or supervised exercise may be necessary in the presence of unstable cardiovascular symptoms, severe metabolic decompensation, significant musculoskeletal limitation, or other high-risk conditions (117, 118). For those with very low physical activity or prolonged sedentary time, small and achievable behavioral changes, such as step-count goals, short postprandial walks, standing breaks, or gradual replacement of sedentary time with light activity, may represent a practical starting point (98, 119).

### 5.3 Behavioral modification, adherence support, and multimodal care

Despite the central role of lifestyle management in PMOS care, long-term adherence remains a major challenge (13). Reported barriers include fatigue, low mood, anxiety, depression, weight stigma, negative body image, low self-efficacy, insufficient social support, lack of reliable information, limited access to professional guidance, time constraints, and inadequate follow-up (107, 120, 121).

Inconsistent online health information may further undermine adherence. Many women with PMOS seek lifestyle advice from internet sources, where recommendations on diet, exercise, supplements, and weight loss may be variable in quality and not always evidence-based (122). Healthcare professionals should therefore provide clear, non-stigmatizing, and evidence-informed counseling, while helping patients evaluate online information critically (120–122).

Behavioral strategies can support long-term lifestyle change. SMART goals, self-monitoring, feedback, motivational interviewing, problem-solving, relapse-prevention planning, and positive reinforcement may help patients translate general recommendations into achievable actions (27, 123). Examples include setting a weekly walking target, scheduling two resistance-training sessions per week, reducing sedentary time by a defined amount, or using step-count and activity-break reminders. These goals should be realistic, revisited regularly, and adjusted according to progress, symptoms, and patient preference (13, 27).

Psychological support is an important component of individualized care. Cognitive-behavioral approaches and broader health behavioral interventions may improve self-efficacy, coping skills, anxiety, depressive symptoms, and maintenance of healthy behaviors (97, 124). Social support from family members, peers, patient groups, and healthcare teams may further improve motivation and reduce isolation (121, 125). Digital health tools, including mobile applications and wearable devices, may facilitate education, self-monitoring, feedback, peer support, and remote follow-up, although their long-term effectiveness and equity of access require further evaluation (126, 127).

Because PMOS involves metabolic, reproductive, dermatologic, and psychological dimensions, multimodal management is often needed. Combining dietary intervention, exercise, sedentary behavior reduction, behavioral support, sleep management, and psychological care may produce broader benefits than a single-component intervention, particularly for women with obesity, insulin resistance, mood symptoms, or reproductive goals (25, 27, 128). Complementary approaches such as mindfulness, meditation, and yoga may improve stress, quality of life, and adherence for some patients, but stronger evidence is needed before they can be recommended as stand-alone treatments (108).

Lifestyle intervention should also be integrated with pharmacological and reproductive management when clinically indicated (13, 95). Depending on symptoms, metabolic risk, reproductive plans, and contraindications, women with PMOS may receive metformin, combined oral contraceptives, anti-androgen therapy, ovulation induction agents, or weight-management pharmacotherapy. Exercise and sedentary behavior reduction may complement these treatments by improving fitness, insulin sensitivity, body composition, mental health, and long-term health behaviors (25, 27, 77). Future studies should report medication use consistently and evaluate whether pharmacological therapy modifies responses to lifestyle intervention (25, 27).

Multidisciplinary care can support individualized and sustainable management (128). Collaboration among gynecologists, endocrinologists, primary care physicians, dietitians, exercise professionals, psychologists, nurses, and reproductive specialists may help deliver coordinated assessment, realistic prescriptions, behavioral support, and follow-up. However, multidisciplinary care should be implemented in a feasible and accessible manner, with attention to cost, referral pathways, patient preference, and health-system resources (121).

Overall, behavioral and psychological support should be embedded within lifestyle care to improve feasibility, reduce attrition, and support long-term maintenance. Multimodal and multidisciplinary approaches may be particularly useful for patients with complex metabolic, reproductive, dermatologic, or psychological needs. Because direct evidence on the optimal combination, sequencing, and phenotype-specific tailoring of lifestyle components remains limited, these recommendations should be interpreted as pragmatic clinical considerations rather than definitive phenotype-specific prescriptions (25, 27).

## 6 Challenges, clinical implementation, and future directions

Although physical activity and exercise are established components of PMOS lifestyle management, sedentary behavior reduction is an emerging target with a comparatively limited PMOS-specific evidence base. Translation into routine clinical practice remains constrained by methodological heterogeneity, limited long-term trial data, adherence barriers, insufficient personalization, and health-system constraints.

### 6.1 Evidence gaps and methodological challenges

A major limitation of the current literature is the scarcity of large, long-term randomized controlled trials evaluating exercise and sedentary behavior interventions in women with PMOS (13, 27). Many existing trials have relatively small sample sizes, short intervention periods, variable diagnostic criteria, and heterogeneous exercise prescriptions. Exercise modality, intensity, frequency, session duration, intervention length, supervision, adherence monitoring, and co-interventions such as diet or pharmacotherapy differ substantially across studies, limiting comparability and making it difficult to define a single optimal exercise prescription (26, 27). The comparative effects of aerobic exercise, resistance training, combined training, and HIIT remain uncertain, although available evidence suggests that these modalities can improve insulin resistance, body composition, cardiorespiratory fitness, and selected hormonal markers (26, 31). Future studies should report exercise protocols in sufficient detail, including intensity prescription, progression, adherence, supervision, and adverse events, to support reproducibility and clinical translation.

Evidence on sedentary behavior is even more limited. Most PMOS-specific studies are observational or based on modeled substitution analyses, and few trials have tested sedentary behavior reduction as an independent intervention (18, 129). Therefore, the causal effects of reducing total sedentary time or interrupting prolonged sitting on insulin resistance, adiposity, lipid metabolism, reproductive outcomes, and quality of life remain uncertain. Future studies should use objective measures such as accelerometry, distinguish sedentary time from lack of moderate-to-vigorous physical activity, and evaluate practical strategies such as timed activity breaks, standing or walking prompts, environmental modifications, and digital reminders (130, 131).

Another important gap is the limited assessment of long-term clinical outcomes. Most exercise trials focus on intermediate outcomes such as HOMA-IR, fasting insulin, BMI, waist circumference, lipid profiles, and androgen levels (26, 31). Fewer studies assess long-term cardiometabolic outcomes, NAFLD progression, cardiovascular events, fertility, pregnancy outcomes, live birth, or sustained quality-of-life improvement (15, 132). Future trials should include both metabolic and reproductive endpoints and should evaluate whether lifestyle interventions modify long-term risk trajectories in different PMOS phenotypes (8, 89).

### 6.2 Adherence, psychological barriers, and patient-centered care

Low adherence remains one of the central barriers to effective lifestyle intervention in PMOS. Only a subset of women with PMOS meet recommended physical activity levels, and prolonged sedentary behavior is common (77, 133). Barriers include weight stigma, depression, anxiety, low self-efficacy, fatigue, lack of social support, limited access to supervised exercise, competing work or caregiving responsibilities (113, 134).

These barriers highlight the need for patient-centered lifestyle counseling rather than generic advice. Behavioral strategies such as shared goal-setting, self-monitoring, feedback, motivational interviewing, peer support, and family involvement may improve adherence and long-term maintenance (123, 135, 136). Psychological assessment and support are also important because anxiety, depression, body image concerns, and weight stigma may reduce motivation and participation. Lifestyle intervention should therefore be framed around metabolic health, reproductive goals, mental health, physical function, and quality of life rather than weight loss alone (97, 137).

Supervised and group-based programs may improve adherence for some patients, but they may not be feasible for all. Hybrid approaches combining clinical guidance, home-based exercise, remote coaching, wearable activity tracking, and periodic follow-up may offer a practical compromise. Future research should evaluate which delivery models are most acceptable, scalable, cost-effective, and sustainable for different patient groups (127, 138, 139).

### 6.3 Individualized and multimodal management

Individualization is essential because women with PMOS differ in phenotype, BMI, insulin resistance, hyperandrogenism, reproductive goals, cardiometabolic risk, psychological burden, physical fitness, musculoskeletal limitations, and personal preferences (6, 113). Rather than applying phenotype-based prescriptions rigidly, clinicians should use PMOS phenotype and metabolic profile to inform risk assessment and shared decision-making (13, 103).

For women with overweight or obesity and insulin resistance, exercise programs may emphasize gradual progression toward moderate-to-vigorous aerobic activity combined with resistance training, alongside dietary management and behavioral support (25, 77). For lean women with PMOS, the focus should not be weight loss alone; exercise may be prescribed to improve insulin sensitivity, cardiorespiratory fitness, menstrual regularity, mental health, and long-term cardiometabolic risk (14, 77, 113). For women with severe obesity, comorbidities, low baseline fitness, or musculoskeletal pain, lower-intensity starting points, supervised progression, and resistance or low-impact aerobic options may improve safety and adherence (140, 141).

Sedentary behavior reduction should be integrated into daily routines (18, 98). Practical strategies may include standing or walking breaks every 30–60 minutes, brief postprandial walking, active commuting when feasible, reducing recreational screen time, using reminders, and restructuring work or study environments to interrupt prolonged sitting. These strategies should be presented as an adjunct to physical activity and exercise rather than a substitute for structured exercise (98, 142, 143).

Lifestyle intervention should be coordinated with pharmacological and reproductive management when clinically indicated, because many women with PMOS receive metformin, combined oral contraceptives, anti-androgen therapy, ovulation induction agents, or weight-management pharmacotherapy depending on symptoms, metabolic risk, reproductive plans, and contraindications (95). Medication use should be reported consistently in future trials to clarify whether pharmacological treatment modifies responses to lifestyle interventions (27).

Multidisciplinary care may improve implementation. Collaboration among endocrinologists, gynecologists, reproductive specialists, dietitians, exercise professionals, psychologists, nurses, and primary care providers can support more comprehensive management (97, 121). However, multidisciplinary models require training, referral pathways, time, reimbursement structures, and accessible patient education materials. Strengthening healthcare provider training in lifestyle counseling and behavioral change techniques may improve the quality and consistency of care (121).

### 6.4 Public health and policy implications

At the public health level, improving awareness of PMOS and its metabolic-reproductive implications may facilitate earlier diagnosis and more effective lifestyle support (144–146). Educational resources should provide evidence-based information on physical activity, exercise, sedentary behavior, nutrition, mental health, and reproductive health while addressing misinformation commonly encountered online (22, 24).

Community, workplace, and school environments can also influence physical activity and sedentary behavior (147–149). Accessible exercise spaces, active transport opportunities, structured community programs, and environments that encourage regular movement breaks may support healthier behaviors. However, public health strategies should avoid stigmatizing weight-focused messaging and should emphasize inclusive, feasible, and sustainable approaches (22, 24).

Policy efforts should support integration of PMOS lifestyle management into chronic disease prevention and reproductive health services (150, 151). Future implementation research should assess feasibility, cost-effectiveness, equity, and long-term outcomes of digital health programs, supervised exercise referral pathways, multidisciplinary care, and sedentary behavior reduction interventions.

In summary, physical activity and exercise are central components of PMOS management, and sedentary behavior reduction is a promising adjunctive strategy. Current evidence supports benefits for several intermediate metabolic outcomes, but stronger evidence is needed for long-term cardiometabolic outcomes, reproductive endpoints, sedentary behavior interventions, and phenotype-specific implementation. Future research should prioritize adequately powered long-term trials, objective measurement of physical activity and sedentary behavior, standardized reporting of exercise prescriptions, evaluation of combined lifestyle-pharmacological approaches, and patient-centered strategies that address adherence, psychological health, equity, and real-world feasibility (89, 152, 153).

## 7 Conclusions

PMOS-related metabolic-reproductive dysfunction arises from interconnected mechanisms involving hyperandrogenism, IR, adipose tissue dysfunction, mitochondrial dysfunction, oxidative stress, chronic low-grade inflammation, neuroendocrine dysregulation, and genetic or epigenetic susceptibility. These pathways contribute not only to metabolic complications such as obesity, dyslipidemia, T2DM risk, NAFLD, and cardiometabolic abnormalities, but also to reproductive features including menstrual irregularity, ovulatory dysfunction, and fertility-related impairment. Current evidence supports physical activity and structured exercise as important components of PMOS management. Aerobic exercise, resistance training, combined training, and HIIT may improve insulin sensitivity, body composition, cardiorespiratory fitness, lipid profiles, inflammatory markers, and selected hormonal or menstrual outcomes. However, intervention effects vary according to baseline BMI, metabolic risk, exercise modality, intensity, duration, supervision, adherence, and co-interventions. Evidence for hard reproductive and cardiovascular outcomes remains limited. Sedentary behavior is associated with adverse metabolic and quality-of-life outcomes in women with PMOS, but its independent causal role remains less certain because most available evidence is observational. Reducing total sedentary time and interrupting prolonged sitting are practical and low-risk strategies that may complement structured exercise, but PMOS-specific randomized trials are needed to determine their independent metabolic and reproductive effects.

Effective lifestyle management should be individualized and patient-centered, taking into account PMOS phenotype, metabolic profile, reproductive goals, psychological health, physical fitness, access to support, and patient preferences. Integration with dietary intervention, pharmacotherapy, behavioral strategies, digital tools, and multidisciplinary care may improve adherence and long-term sustainability. Future research should focus on large-scale long-term trials, standardized exercise reporting, objective sedentary behavior assessment, combined lifestyle-pharmacological approaches, and precision medicine frameworks. Translating these findings into accessible clinical and public health strategies may improve metabolic, reproductive, psychological, and quality-of-life outcomes for women with PMOS.
